# Pain Experience is Somatotopically Organized and Overlaps with Pain Anticipation in the Human Cerebellum

**DOI:** 10.1007/s12311-018-0930-9

**Published:** 2018-02-26

**Authors:** F. H. S. Michelle Welman, Albertine E. Smit, Joost L. M. Jongen, Dick Tibboel, Jos N. van der Geest, Jan C. Holstege

**Affiliations:** 1 Department of Neuroscience, Erasmus MC, Wytemaweg 80, 3015 CN Rotterdam, the Netherlands; 2 Department of Neurology, Erasmus MC, Room G3-78, Groene Hilledijk 301, 3075 EA Rotterdam, the Netherlands; 3 Department of Intensive Care and Pediatric Surgery, Erasmus MC, Wytemaweg 80, 3015 CN Rotterdam, the Netherlands

**Keywords:** fMRI, Nociceptive stimulation, Anticipation, Electrical, Cerebellum, Pain matrix

## Abstract

Many fMRI studies have shown activity in the cerebellum after peripheral nociceptive stimulation. We investigated whether the areas in the cerebellum that were activated after nociceptive thumb stimulation were separate from those after nociceptive toe stimulation. In an additional experiment, we investigated the same for the anticipation of a nociceptive stimulation on the thumb or toe. For his purpose, we used fMRI after an electrical stimulation of the thumb and toe in 19 adult healthy volunteers. Following nociceptive stimulation, different areas were activated by stimulation on the thumb (lobule VI ipsilaterally and Crus II mainly contralaterally) and toe (lobules VIII-IX and IV-V bilaterally and lobule VI contralaterally), i.e., were somatotopically organized. Cerebellar areas innervated non-somatotopically by both toe and thumb stimulation were the posterior vermis and Crus I, bilaterally. In the anticipation experiment, similar results were found. However, here, the somatotopically activated areas were relatively small for thumb and negligible for toe stimulation, while the largest area was innervated non-somatotopically and consisted mainly of Crus I and lobule VI bilaterally. These findings indicate that nociceptive stimulation and anticipation of nociceptive stimulation are at least partly processed by the same areas in the cerebellum. This was confirmed by an additional conjunction analysis. Based on our findings, we hypothesize that input that is organized in a somatotopical manner reflects direct input from the spinal cord, while non-somatotopically activated parts of the cerebellum receive their information indirectly through cortical and subcortical connections, possibly involved in processing contextual emotional states, like the expectation of pain.

**Electronic supplementary material:**

The online version of this article (10.1007/s12311-018-0930-9) contains supplementary material, which is available to authorized users.

## Introduction

The experience of pain is produced by a complex neuronal system, which involves nociception, i.e., the signaling of tissue damage, as well as various cognitive and emotional components that are inherent to pain as a feeling [1]. One of the subcortical structures of the pain matrix, which is consistently activated after a peripheral nociceptive stimulus, is the cerebellum [2, 3]. Casey and coworkers were one of the first to identify cerebellar activity in the vermis after 50 °C heat stimuli of 5 s’ duration on the forearm using PET imaging [4]. Since then, many imaging studies have confirmed cerebellar activity after electrical, laser, capsaicin, or other types of nociceptive stimulation, mainly involving the vermis (in lobules IV–V), the ipsilateral cortex (lobules IV–VI, Crus I), and the contralateral cortex (lobule VI, Crus I) of the cerebellum [3]. Animal studies that focused on the cerebellum in pain processing have suggested spinally projecting multisensory information from the skin, including tactile Aβ- and nociceptive Aδ- and C-fiber input [5–7]. However, the functional role of the cerebellum in pain processing remains largely unclear. The most widely accepted idea is that cerebellar activity is related to the fine-tuning of the motor output when we experience pain, in order to protect it from further harm [8, 9]. However, a role in pain anticipation [10–12], in the inhibition of pain [13–17], and in perceiving pain induced in others [18] has also been suggested.

Taken together, the abovementioned data strongly indicate that the cerebellum is involved in pain perception, but there are still many questions about the precise nature of this involvement. One of these questions is whether the cerebellum is involved in modulating the processing of the incoming nociceptive signals or with the preparation and execution of a motor response to the nociceptive signal [19]. Another question regarding the role of the cerebellum in pain processing is whether it is instrumental in producing tasks that are localization independent, like pain inhibition and the production of warning signals, or whether it is involved in the precise localization and precise movement planning in response to the nociceptive signals. In the latter case, a more detailed processing of the pain signal will be necessary, that would require a precise somatotopical organization. The present fMRI study was initiated to investigate whether activation in the cerebellum after nociceptive stimulation is somatotopically organized. In addition, to learn more about the nature of cerebellar processing, we performed a separate study on the somatotopic activation pattern in the cerebellum during the anticipation of a nociceptive stimulus. Finally, we determined to which extend the cerebellar areas activated by nociceptive stimulation were separate from those activated by the anticipation of such stimuli.

## Methods

The pain-only and pain anticipation experiments were approved by the medical ethics committee of Erasmus MC. All subjects had given written informed consent prior to the study.

### Nociceptive Stimulation

Nociceptive stimuli were administered by transcutaneous electrical stimulation (5-Hz sine waves with adjustable intensity) using a Neurometer (Neurotron Inc., Baltimore, MD). The onset and offset of the stimuli were triggered manually.

Prior to scanning, subjects had to verbally rate the pain stimulation on a Numerical Rating Scale (NRS) from 1 (no pain) to 10 (unbearable pain). The intensity of the electrical stimulus was individually adjusted to elicit a stimulus that was rated as seven.

### Pain-Only Experiment

Seventeen healthy subjects (10 males, 7 females; age range 18 to 29 years) participated in the pain study. One male subject was excluded due to technical problems.

We used a block design paradigm in which resting periods alternated with stimulation periods. During a stimulation period, the subject received a nociceptive stimulus to the right thumb and right big toe. Volunteers underwent five fMRI scans, each consisting of five blocks of nociceptive thumb stimulation and five blocks of nociceptive big toe stimulation. Stimulus blocks lasted 15 s; resting periods had three different durations: 10, 15, and 20 s. The order of the location of stimulation was random to limit a possible effect due to anticipation. Every scan started and ended with a resting period of 20 s.

### Pain Anticipation Experiment

Seventeen healthy subjects (10 males, 7 females; age range 20 to 30 years) participated in the pain anticipation study. These included the same subjects that also took part in the pain-only experiment, except two that were replaced by two other volunteers. Three subjects were excluded (two males, one female) due to incomplete data.

We used a block design with three conditions: rest, nociceptive anticipation, and nociceptive stimulation. Identical to the pain-only experiment, the nociceptive stimulus was given on either the right thumb or the right big toe. Volunteers underwent four fMRI scans, each consisting of 4 cycles of blocks of rest and nociceptive anticipation, followed by nociceptive stimulation in both thumb and big toe. During nociceptive anticipation blocks, subjects were shown a red screen to indicate that nociceptive thumb stimulation was imminent and a blue screen to indicate the same for big toe stimulation. Resting periods had two different durations: 20 and 30 s; nociceptive stimulus blocks lasted 10 s; anticipation blocks had two different durations: 10 and 15 s. The order of the location of stimulation was random. Every scan started and ended with a resting period of 20 s.

### Data Acquisition

Data were acquired on a 3T MRI scanner (HD platform, GE Healthcare, Milwaukee, WI) using a dedicated eight-channel head coil. For anatomical reference a 192-slice high-resolution three-dimensional inversion recovery (IR) fast spoiled gradient echo (FSPGR) T1-weighted image was acquired (parameters: slice thickness 1.6 mm with 0.8-mm overlap; repetition time (TR)/echo time (TE)/inversion time (TI) 10.3/2.0/300 ms; 18° flip angle; matrix 416 × 256 and field of view (FOV) 250 × 180 mm^2^). For the functional scans, a 32-slice single-shot T2*-weighted echo-planar imaging (EPI) sequence sensitive to blood oxygenation level dependent (BOLD) contrast was used (parameters: slice thickness 3.0 mm and a 0.5-mm gap; TR/TE 2500/30 ms; 75° flip angle; 64 × 96 matrix with a FOV of 220 × 220 mm^2^; voxel sizes 3.0 × 3.4 × 2.9 mm^3^). Acquisition time of each scan was 6 min 50 s, including 10 s of dummy scans that were discarded.

### Data Analysis

The functional imaging data were pre-processed and analyzed using the statistical parametric mapping toolbox (SPM 5, Wellcome Department of Cognitive Neurology, London, UK) run with MATLAB (version 7.8, Mathworks, Sherborn, MA). The anatomical scans were segmented into maps for white matter, grey matter, and cerebrospinal fluid. Normalization into the Montreal Neurological Institute (MNI) space was performed with parameters obtained during segmentation. The anatomical data were re-sliced into voxels of 1 × 1 × 1 mm^3^.

Functional scans were re-aligned, coregistered to the grey matter map, normalized with parameters obtained during segmentation, and re-sliced into 2 × 2 × 2-mm^3^ voxels and subsequently smoothed with a Gaussian kernel of 6-mm FWHM (full width at half maximum).

Single subject statistical analysis was performed with the general linear model. The fMRI time series was modeled as a series of event blocks convolved with a canonical hemodynamic response function. The two conditions of the pain study were nociceptive stimulation of the thumb and nociceptive stimulation of the big toe. There were four conditions of the anticipation experiment: anticipation of nociceptive stimulation of the thumb, anticipation of nociceptive stimulation of the big toe, actual nociceptive stimulation of the thumb, and actual nociceptive stimulation of the big toe. In addition, the time derivatives were modeled and movement parameters were included as regressors of no interest. The model was estimated with a high-pass filter with a cutoff period of 128 s. For each session, a T-contrast map was calculated for each condition, which was used in the second level, random effects analysis.

First, whole brain group results for thumb stimulation and toe stimulation were evaluated. For this analysis, an a priori statistical threshold of *p* < 0.001 at the voxel level (uncorrected) and family wise error rate (FWER) correction (*p* < 0.05) at the group level was used, resulting in a minimum cluster extent of 104 voxels. Anatomical structures were defined with the Talairach Daemon Labels atlas of the WFU Pick Atlas [20] in AAL (Automated Anatomical Labeling) [21], to aid with the description of the whole brain analysis results.

Further analysis focused on the cerebellum. The cerebellum was isolated and normalized to a cerebellum-specific template using the SUIT procedure, which provides a widely used analysis method for cerebellar fMRI data [9, 22, 23]. Subsequently, the unsmoothed functional data was modeled as described previous. Finally, the contrast images were smoothed with a Gaussian kernel of 6-mm FWHM. Anatomical structures were labeled according to the standard AAL atlas according to the MNI coordinates of the observed activation patterns.

To visualize differences in the activation induced by thumb and toe stimulation, activation maps were made for the thumb > toe and the toe > thumb stimulations. Differences in the activation induced by thumb and toe anticipation were visualized with activation maps of anticipation thumb > anticipation toe and anticipation toe > anticipation thumb. Conjunction analysis was performed to evaluate the overlap in activation after thumb and toe stimulation as well as thumb and toe anticipation. Comparisons used to study differences in activation included activation tables of thumb pain > thumb anticipation, thumb anticipation > thumb pain, toe pain > toe anticipation and toe anticipation > toe pain. Areas both active during anticipation of pain and sensation of pain were studied with conjunction analysis for thumb and toe. Overall conjunction was studied with a full factorial conjunction analysis with thumb pain, toe pain, thumb anticipation, and toe anticipation. For these analyses, an a priori threshold of *p* < 0.005 (uncorrected) and a minimum cluster extent of 24 voxels were used, based on a similar cerebellar fMRI study by Coombes and Misra [9], in which an empirical cluster-extent threshold of 192 mm^3^ was determined, corresponding to 24 2 × 2 × 2-mm voxels. The cluster-extent threshold of 24 voxels for the cerebellar subregions was based on the assumption that areas of true neural activity will tend to stimulate signal changes over contiguous voxels [24]. Anatomical structures were defined with the standard AAL atlas in AAL [21].

## Results

All participants were stimulated on the right thumb and the right big toe. They generally described the stimulation as a painful sensation, a touch-like sensation was never reported. NRS pain scores were obtained after every session resulting in a mean score of 7.4 (± 0.7 SD) for the thumb and 7.7 (± 0.8 SD) for the big toe in the pain-only experiment and 7.5 (± 0.7 SD) for the thumb and 7.7 (± 0.7 SD) for the big toe in the pain anticipation experiment. The intensity of the stimulus remained constant during the experiment in most subjects. In a few occasions, when the subjective experience of pain increased, the stimulus intensity was lowered and vice versa.

### General Brain Activation After Nociceptive Thumb or Toe Stimulation

**Fig. 1:**
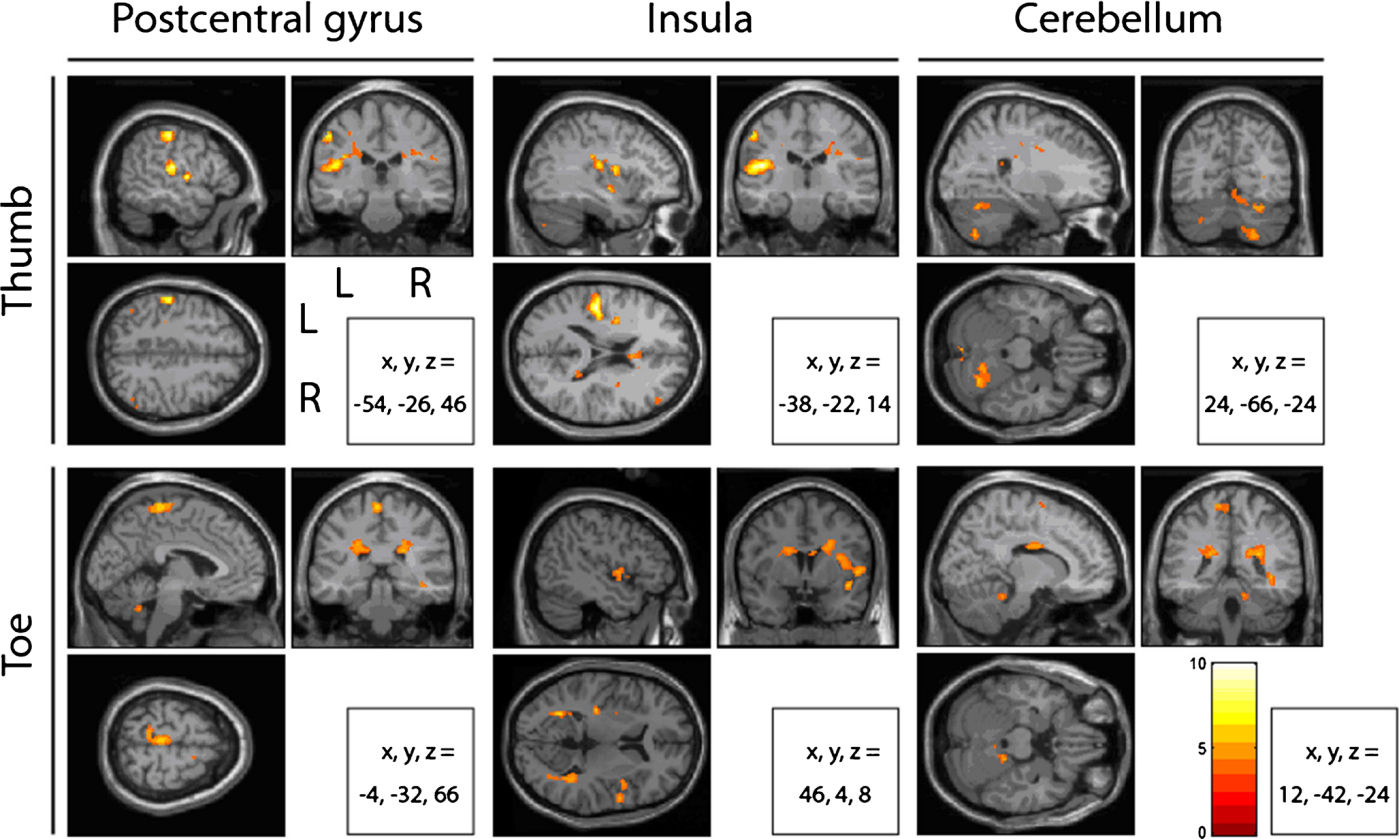
Group analysis maps overlaid on axial, coronal, and sagittal images from a standard MNI brain showing clusters of activation in the post central gyrus, insula, and cerebellum after nociceptive stimulation of the thumb and toe. Clusters are coded from dark red (*T* = 0) to bright yellow (*T* = 10) as indicated by the scale and L = left and R = right. A threshold of *p* = 0.001 was used (*n* = 16)

A group analysis after stimulation of the thumb and big toe yielded activation in various areas of the brain, including the insula, the post central gyrus, and the cerebellum (Supplementary Table S1, Fig. 1). For the thumb, the post central gyrus was activated laterally on the contralateral side, while for the toe activation was found mainly medially. The insula and cingulate cortex were activated bilaterally. These findings are in general agreement with previous studies.

### Cerebellar Activation During Nociceptive Thumb or Toe Stimulation

**Fig. 2:**
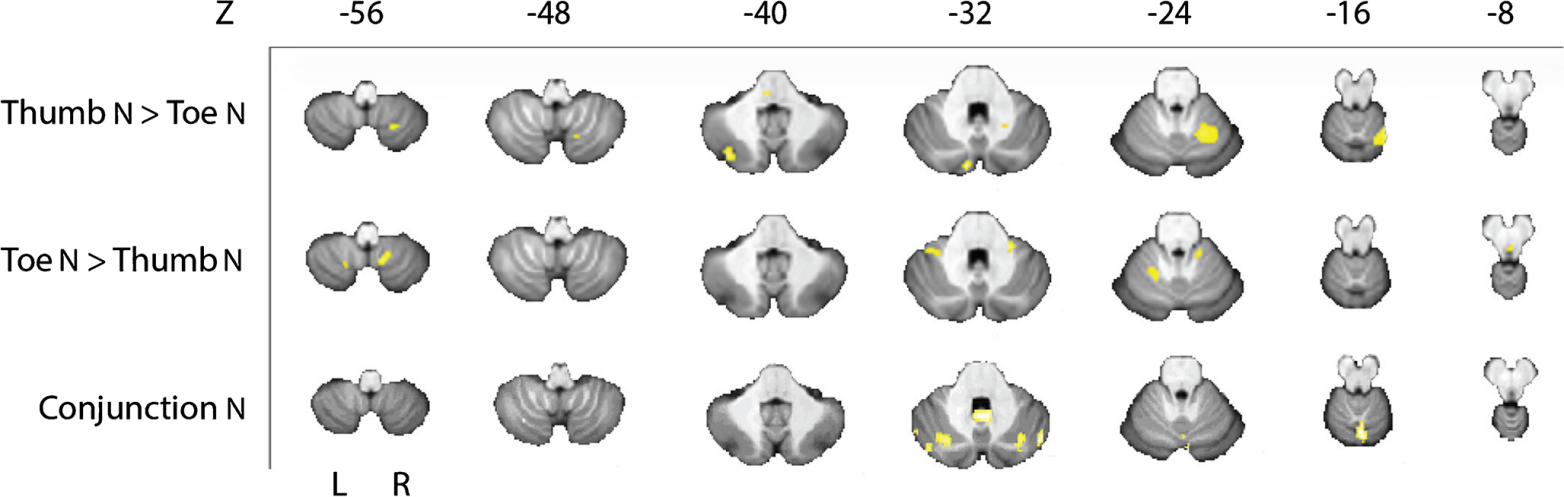
Group analysis of cerebellar data displayed on the SUIT template. The upper two rows show stimulus specific differences in activation after nociceptive stimulation of thumb or toe found with the thumb > toe contrast (first row) and toe > thumb contrast (second row). Conjunction results are shown in the third row and represent the overlap of activation found after nociceptive stimulation of thumb or toe. Slices are arranged from inferior (*Z* = − 56) to superior (*Z* = − 8) and L = left and R = right. A threshold of *p* = 0.005 was used (*n* = 16)

In the cerebellum, we found an activation during thumb and toe stimulation. In order to determine whether this activation was somatotopically organized, a comparison analysis was performed. In several areas, the activation was correlated significantly more with thumb stimulation, than with toe stimulation (thumb > toe, *p* < 0.005; Table 1, Fig. 2). These areas included a relatively large cluster in lobule VI ipsilaterally, in Crus II contralaterally, and a cluster in lobule VIII on the ipsilateral side of the stimulation. Activation that was correlated more with toe than with thumb stimulation (toe > thumb, *p* < 0.005; Table 1, Fig. 2) was located bilaterally in lobules VIII and IX and in lobules IV and V and contralaterally in lobule VI. We also examined which areas in the cerebellum were activated by thumb as well as toe stimulation, i.e., that were not somatotopically organized. For this purpose, we used a conjunction analysis to identify areas where the activation was significantly correlated with both toe and thumb stimulation (conjunction, *p* < 0.005; Table 1, Fig. 2). In this case, relatively strong activation was found in the vermis at the levels of lobule IV–VII and IX–X. In addition, activation was found bilaterally in Crus I.

**Table 1:** Group analysis of differences and overlap in activation in the cerebellum after nociceptive stimulation of thumb and toe

Cluster	*T*	*x*	*y*	*z*	Side	*N* voxels	Structure
THUMB > TOE nociceptive stimulation							
28	4.34	− 8	− 84	− 32	Left	28	*Cerebellum_Crus2*
244	4.23	22	− 56	− 18	Right	227	*Cerebellum_6*
					Right	17	*Cerebellum_4_5*
59	4.21	− 34	− 76	− 40	Left	59	*Cerebellum_Crus2*
10	3.51	46	− 76	− 46	Right	10	Cerebellum_Crus2
44	3.44	22	− 62	− 56	Right	44	*Cerebellum_8*
8	3.4	16	− 80	− 52	Right	7	Cerebellum_7b
					Right	1	Cerebellum_Crus2
3	3.09	36	− 70	− 36	Right	3	Cerebellum_Crus1
2	3.05	38	− 76	− 34	Right	2	Cerebellum_Crus1
TOE > THUMB nociceptive stimulation							
33	4.35	12	− 56	− 58	Right	20	*Cerebellum_9*
					Right	13	*Cerebellum_8*
47	4.01	24	− 38	− 30	Right	41	*Cerebellum_4_5*
					Right	6	*Cerebellum_3*
31	3.92	− 34	− 42	− 34	Left	23	*Cerebellum_6*
					Left	8	*Cerebellum_Crus1*
42	3.79	− 18	− 54	− 22	Left	27	*Cerebellum_6*
					Left	15	*Cerebellum_4_5*
53	3.54	− 18	− 66	− 52	Left	53	*Cerebellum_8*
8	3.4	− 4	− 56	− 36	–	5	Vermis_9
						3	Cerebellum_9
CONJUNCTION nociceptive stimulation							
175	3.84	2	− 66	− 14	–	104	*Vermis_6*
					–	38	*Vermis_4_5*
					–	26	*Vermis_7*
					Right	5	*Cerebellum_Crus1*
					Right	2	*Cerebellum_6*
82	3.83	0	− 54	− 32	–	46	*Vermis_9*
					–	23	*Vermis_10*
					Left	7	*Cerebellum_9*
					Right	6	*Cerebellum_9*
4	3.48	− 10	− 34	− 10	Left	4	Cerebellum_4_5
70	3.42	30	− 78	− 30	Right	52	*Cerebellum_Crus1*
					Right	18	*Cerebellum_6*
28	3.38	46	− 70	− 32	Right	28	*Cerebellum_Crus1*
27	3.18	− 28	− 70	− 32	Left	27	*Cerebellum_Crus1*
11	3.14	− 34	− 64	− 52	Left	7	Cerebellum_8
29	3.1	− 42	− 76	− 34	Left	27	*Cerebellum_Crus1*
					Left	2	*Cerebellum_Crus2*
11	3.07	− 52	− 64	− 34	Left	11	Cerebellum_Crus1
4	2.84	− 50	− 60	− 24	Left	4	Cerebelum_Crus1
3	2.79	50	− 62	− 28	Right	3	Cerebellum_Crus1

Cluster size (Cluster) in voxels, *T* max (*T*) and its MNI coordinates (*x*, *y*, *z*), and side at which the activation occurs (Side) are given followed by the specification of the number of voxels (*N* voxels) per structure (Structure) within that cluster. Significant areas with an uncorrected threshold of *p* = 0.005 and a cluster-extent of at least 24 voxels are shown in italic, while areas containing subthreshold clusters are shown in regular face (*n* = 16)

### Cerebellar Activation During Anticipation of Nociceptive Thumb or Toe Stimulation

In a separate experiment, we examined the activation in the cerebellum during the anticipation of a nociceptive stimulus to the thumb or the big toe. This experiment also included a nociceptive experience after the anticipation phase, which showed a similar activation pattern in the whole brain as we found in the first experiment (described previously), including activation clusters in the (contralateral) somatosensory cortex, the insula, the cingulate cortex, and the cerebellum (details not shown). The detailed analysis of the cerebellar contrasts was similar to the results of the first experiment (compare Supplementary Table S2 with Table 1).

**Fig. 3:**
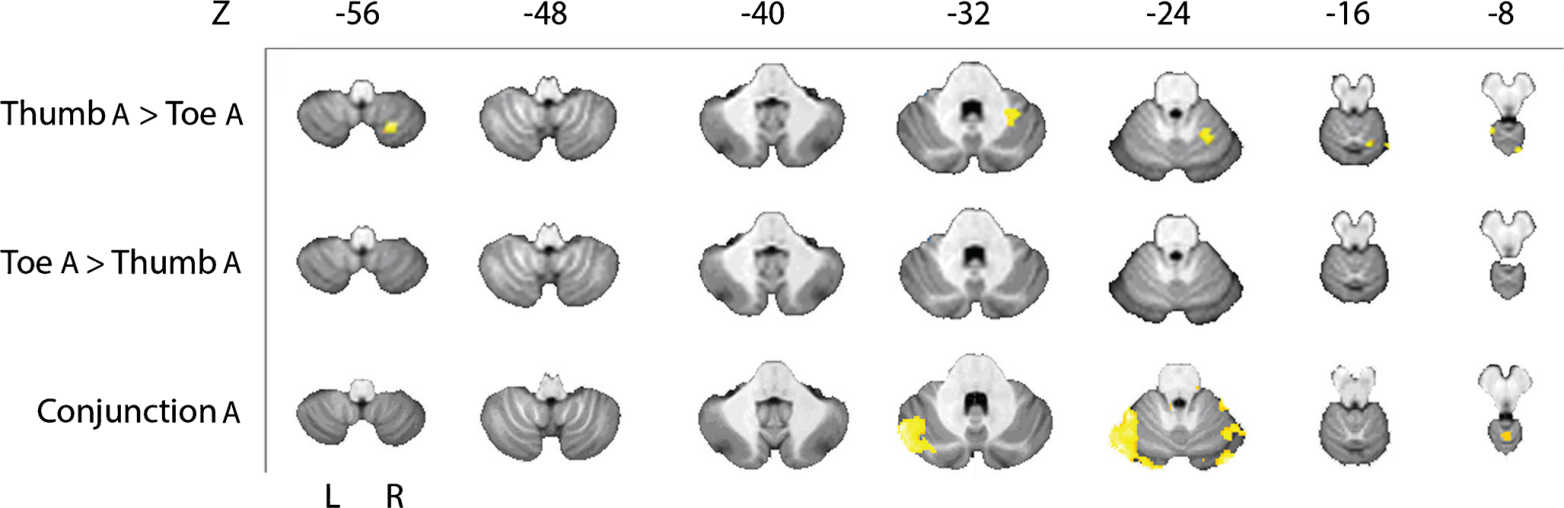
Group analysis of cerebellar data displayed on the SUIT template. The upper two rows show stimulus specific differences in activation after anticipation of nociceptive stimulation of thumb or toe found with the thumb > toe contrast (first row) and toe > thumb contrast (second row). Conjunction results are shown in the third row and represent the overlap of activation found after nociceptive stimulation of thumb or toe. Slices are arranged from inferior (*Z* = − 56) to superior (*Z* = − 8) and L = left and R = right. A threshold of *p* = 0.005 was used (*n* = 14)

We then examined cerebellar activation during the anticipation phase. We found a bilateral activation in the cerebellum during the anticipation of nociceptive stimulation of the thumb or toe. In order to determine whether this organization was somatotopically organized, a comparison analysis was performed. We identified areas that were correlated significantly more with the anticipation of thumb stimulation, than with that of toe stimulation (thumb > toe, *p* < 0.005; Table 2, Fig. 3). The activated areas were in lobules VI and VIII ipsilaterally. No clusters were identified that were correlated significantly more with anticipation of nociceptive toe than thumb stimulation, after cluster-extent-based thresholding (toe > thumb, *p* < 0.005; Table 2, Fig. 3). We then analyzed conjunction, i.e., areas where the activation was significantly correlated with the anticipation of toe as well as with the anticipation of thumb stimulation. Activation was found mainly in Crus I and in lobule VI bilaterally (conjunction, *p* < 0.005; Table 2, Fig. 3).

**Table 2:** Group analysis of differences and overlap in activation in the cerebellum after anticipation of nociceptive stimulation of thumb and toe

Cluster	*T*	*x*	*y*	*z*	Side	*N* voxels	Structure
THUMB > TOE anticipation of pain							
2	6.38	− 18	− 42	− 4	Left	2	Cerebellum_4_5
47	4.46	22	− 64	− 58	Right	47	*Cerebellum_8*
105	4.13	38	− 48	− 28	Right	105	*Cerebellum_6*
22	3.92	10	− 66	− 8	Right	9	Cerebellum_6
					Right	6	Cerebellum_4_5
					–	5	Vermis_4_5
					–	2	Vermis_6
4	3.54	12	− 50	− 12	Right	4	Cerebellum_4_5
2	3.38	20	− 72	− 46	Right	2	Cerebellum_8
TOE > THUMB anticipation of pain							
8	3.82	52	− 72	− 36	Right	8	Cerebellum_Crus1
1	3.56	− 32	− 42	− 44	Left	1	Cerebellum_8
CONJUNCTION anticipation of pain							
1036	4.99	− 50	− 60	− 28	Left	831	*Cerebellum_Crus1*
					Left	196	*Cerebellum_6*
126	4.08	34	− 80	− 24	Right	114	*Cerebellum_Crus1*
					Right	12	*Cerebellum_6*
51	3.73	34	− 40	− 26	Right	39	*Cerebellum_6*
					Right	12	*Cerebellum_4_5*
74	3.43	38	− 66	− 22	Right	58	*Cerebellum_6*
					Right	16	*Cerebellum_Crus1*
15	3.09	0	− 54	− 8	–	15	Vermis_4_5
15	2.97	− 2	− 48	− 22	–	5	Vermis_3
					Left	5	Cerebellum_3
					–	4	Vermis_4_5
					–	1	Vermis_1_2
2	2.85	10	− 48	− 36	Right	2	Cerebellum_9
1	2.82	14	− 30	− 24	Right	1	Cerebellum_3

Cluster size (Cluster) in voxels, *T* max (*T*) and its MNI coordinates (*x*, *y*, *z*), and side at which the activation occurs (Side) are given followed by the specification of the number of voxels (*N* voxels) per structure (Structure) within that cluster. Significant areas with an uncorrected threshold of *p* = 0.005 and a cluster-extent of at least 24 voxels are shown in italic, while areas containing subthreshold clusters are shown in regular face (*n* = 14)

### Comparing Cerebellar Activation During the Experience and the Anticipation of Nociceptive Thumb or Toe Stimulation

**Fig. 4:**
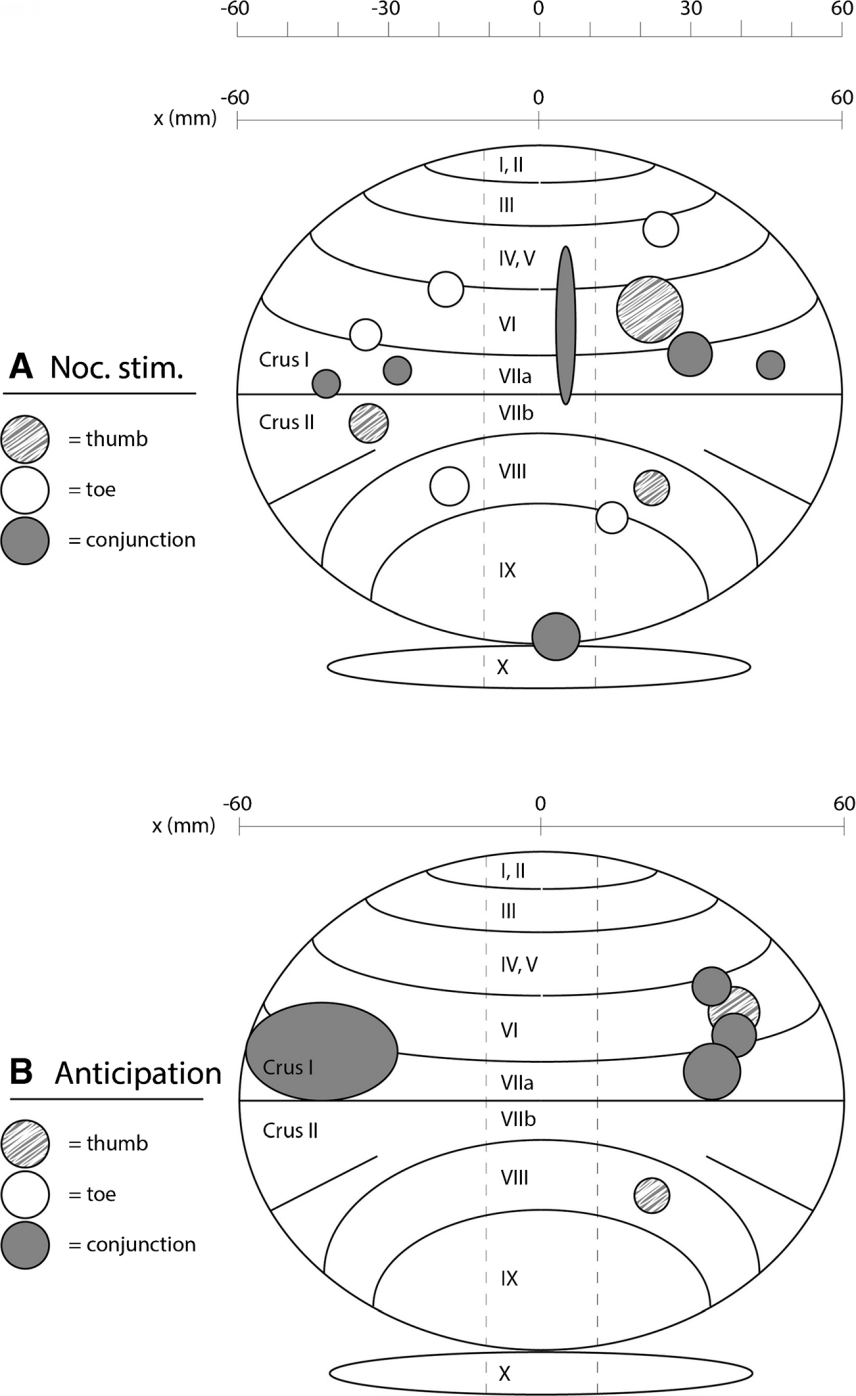
A schematic representation of the cerebellum displaying the results from Tables 1, 2, 3, and 4. The cross section in the sagittal plane in **a** contains cerebellar activation induced by nociceptive stimulation; **b** contains cerebellar activation induced by anticipation of nociceptive stimulation. In both parts, the thumb > toe comparison (thumb; grey/white blobs), toe > thumb comparison (toe; white blobs), and conjunction analysis (conjunction; dark grey blobs) results are shown. The size of the blobs is relative to the number of activated voxels, and the distribution along the horizontal *x*-axis is given for all blobs (from left − 60 mm to right 60 mm)

Since the anticipation experiment included a pain stimulus immediately following the anticipation phase, we were able to compare pain anticipation and pain experience for thumb and toe. Areas in the cerebellum that were activated by pain experience on the thumb but not by pain anticipation were limited in volume and found predominantly in lobule VIII (thumb pain > thumb anticipation, *p* < 0.005; Table 3). In contrast, the total area activated by pain anticipation but not by pain experience was much larger, including lobules IV–V, VI bilaterally, and the vermis (thumb anticipation > thumb pain, *p* < 0.005; Table 3). However, the largest area was found in the conjunction analysis of the thumb (Crus I and lobule VI on both sides (conjunction thumb pain and pain anticipation, *p* < 0.005; Table 3), showing that this area was activated by both pain experience and pain anticipation. For toe stimulation, similar findings were obtained: relatively small areas in the cerebellum were activated by nociceptive toe stimulation and anticipation specifically, while the conjunction analysis showed that a much larger area, located in Crus I and lobule VI bilaterally, was activated by both (*p* < 0.005; Table 4). Since the conjunction analysis of the thumb and toe showed very similar areas of activation, we performed an additional conjunction analysis (*p* < 0.005; Table 5) to identify the areas of the cerebellum that were activated by the nociceptive thumb stimulation, the nociceptive toe stimulation, the anticipation of nociceptive thumb stimulation, and the anticipation of nociceptive toe stimulation. This analysis confirmed that the same areas in Crus I and lobule VI, mostly on the contralateral side, were activated by each of these four types of stimulation. A schematic representation of the results is presented in Fig. 4.

**Table 3:** Group analysis of differences and overlap in activation in the cerebellum after nociceptive stimulation and anticipation of pain of thumb

Cluster	*T*	*x*	*y*	*z*	Side	*N* voxels	Structure
THUMB nociceptive stimulation > THUMB anticipation of pain							
77	3.94	26	− 66	− 52	Right	77	*Cerebellum_8*
23	3.83	16	− 76	− 54	Right	20	Cerebellum_8
					Right	3	Cerebellum_7b
7	3.49	− 8	− 88	− 30	Left	7	Cerebellum_Crus2
15	3.47	− 22	− 78	− 50	Left	15	Cerebellum_7b
3	3.11	20	− 64	− 20	Right	3	Cerebellum_6
5	3.1	26	− 58	− 22	Right	5	Cerebellum_6
1	3.03	16	− 68	− 18	Right	1	Cerebellum_6
THUMB anticipation of pain > THUMB nociceptive stimulation							
578	5.78	− 24	− 48	− 28	Left	333	*Cerebellum_4_5*
					Left	274	*Cerebellum_6*
					Left	13	*Cerebellum_3*
					Left	7	*Cerebellum_Crus1*
229	5.67	26	− 36	− 22	Right	71	*Cerebellum_4_5*
					Right	57	*Cerebellum_Crus2*
					Right	55	*Cerebellum_6*
					Right	46	*Cerebellum_Crus1*
276	5.15	8	− 42	− 14	–	115	*Vermis_3*
					Left	51	*Cerebellum_4_5*
					Right	45	*Cerebellum_4_5*
					Right	43	*Cerebellum_3*
					Left	22	*Cerebellum_3*
31	4.53	− 14	− 44	0	Left	31	*Cerebellum_4_5*
2	3.73	− 8	− 66	− 32	Left	2	Cerebellum_8
15	3.57	− 12	− 52	− 56	Left	15	Cerebellum_9
18	3.42	− 2	− 42	− 36	–	13	Vermis_10
6	3.29	52	− 60	− 50	Right	6	Cerebellum_Crus2
2	3.21	14	− 30	− 24	Right	2	Cerebellum_3
4	3.14	− 12	− 32	− 22	Left	4	Cerebellum_3
3	3.12	52	− 70	− 40	Right	3	Cerebellum_Crus2
1	3.03	14	− 48	− 50	Right	1	Cerebellum_9
CONJUNCTION THUMB nociceptive stimulation and anticipation of pain							
269	5.04	38	− 80	− 24	Right	154	*Cerebellum_Crus1*
					Right	115	*Cerebellum_6*
717	4.08	− 42	− 72	− 20	Left	617	*Cerebellum_Crus1*
					Left	77	*Cerebellum_6*
					Left	23	*Cerebellum_Crus2*
22	3.35	22	− 78	− 28	Right	22	Cerebellum_Crus1
6	2.84	2	− 56	− 6	–	6	Vermis_4_5
1	2.8	36	− 60	− 22	Right	1	Cerebellum_6

Cluster size (Cluster) in voxels, *T* max (*T*) and its MNI coordinates (*x*, *y*, *z*), and side at which the activation occurs (Side) are given followed by the specification of the number of voxels (*N* voxels) per structure (Structure) within that cluster. Significant areas with an uncorrected threshold of *p* = 0.005 and a cluster-extent of at least 24 voxels are shown in italic, while areas containing subthreshold clusters are shown in regular face (*n* = 14)

**Table 4:** Group analysis of differences and overlap in activation in the cerebellum after nociceptive stimulation and anticipation of pain of toe

Cluster	*T*	*x*	*y*	*z*	Side	*N* voxels	Structure
TOE nociceptive stimulation > TOE anticipation of pain							
75	5.78	10	− 66	− 6	–	33	*Vermis_4_5*
					Right	28	*Cerebellum_4_5*
					–	14	*Vermis_6*
6	3.44	− 24	− 78	− 50	Left	6	Cerebellum_7b
2	3.38	36	− 84	− 42	Right	2	Cerebellum_Crus2
4	3.21	0	− 54	− 6	–	4	Vermis_4_5
1	3.03	16	− 42	− 22	Right	1	Cerebellum_4_5
TOE anticipation of pain > TOE nociceptive stimulation							
112	5.04	− 22	− 40	− 30	Left	106	*Cerebellum_4_5*
					Left	6	*Cerebellum_6*
31	4.37	− 12	− 32	− 22	Left	16	*Cerebellum_3*
					–	15	*Vermis_1_2*
10	3.97	32	− 42	− 24	Right	10	Cerebellum_6
2	3.41	26	− 32	− 24	Right	2	Cerebellum_4_5
1	3.1	− 38	− 42	− 34	Left	1	Cerebellum_Crus1
CONJUNCTION TOE nociceptive stimulation and anticipation of pain							
679	4.84	− 26	− 80	− 20	Left	526	*Cerebellum_Crus1*
					Left	125	*Cerebellum_6*
					Left	28	*Cerebellum_Crus2*
138	4.12	46	− 62	− 24	Right	75	*Cerebellum_Crus1*
					Right	63	*Cerebellum_6*
102	3.89	34	− 80	− 24	Right	92	*Cerebellum_Crus1*
					Right	10	*Cerebellum_6*
10	3.61	26	− 38	− 30	Right	10	Cerebellum_4_5
14	3.33	14	− 80	− 24	Right	14	Cerebellum_Crus1
4	3.25	10	− 44	− 24	Right	4	Cerebellum_3
5	2.9	0	− 54	− 6	–	5	Vermis_4_5

Cluster size (Cluster) in voxels, *T* max (*T*) and its MNI coordinates (*x*, *y*, *z*), and side at which the activation occurs (Side) are given followed by the specification of the number of voxels (*N* voxels) per structure (Structure) within that cluster. Significant areas with an uncorrected threshold of *p* = 0.005 and a cluster-extent of at least 24 voxels are shown in italic, while areas containing subthreshold clusters are shown in regular face (*n* = 14)

**Table 5:** Group analysis of overlap in activation in the cerebellum after nociceptive stimulation and anticipation of pain of thumb and toe

Cluster	*T*	*x*	*y*	*z*	Side	*N* voxels	Structure
CONJUNCTION nociceptive stimulation and anticipation of pain, thumb and toe							
586	3.84	− 36	− 62	− 24	Left	487	*Cerebellum_Crus1*
					Left	99	*Cerebellum_6*
75	3.81	36	− 80	− 24	Right	68	*Cerebellum_Crus1*
					Right	7	*Cerebellum_6*
37	3.47	46	− 60	− 24	Right	22	*Cerebellum_Crus1*
					Right	15	*Cerebellum_6*
8	2.89	24	− 74	− 28	Right	8	Cerebellum_Crus1
2	2.75	2	− 54	− 6	–	2	Vermis_4_5
2	2.72	40	− 52	− 30	Right	1	Cerebellum_6
					Right	1	Cerebellum_Crus1
1	2.71	18	− 80	− 26	Right	1	Cerebellum_Crus1

Cluster size (Cluster) in voxels, *T* max (*T*) and its MNI coordinates (*x*, *y*, *z*), and side at which the activation occurs (Side) are given followed by the specification of the number of voxels (*N* voxels) per structure (Structure) within that cluster. Significant areas with an uncorrected threshold of *p* = 0.005 and a cluster-extent of at least 24 voxels are shown in italic, while areas containing subthreshold clusters are shown in regular face (*n* = 14)

## Discussion

In this fMRI study, we have investigated the activation in the cerebellum after nociceptive stimulation of the thumb and the toe and after the anticipation of these stimuli. We found that

1. Nociceptive stimulation of the toe and the thumb activated separate areas in the cerebellum, while areas that were activated by toe as well as thumb stimulation were of similar size. This finding shows that the activation of the cerebellum is partly organized in a somatotopic fashion.
2. Anticipation of nociceptive stimuli on the thumb or toe mainly activated the same areas of the cerebellum. Much smaller areas were activated by the anticipation of thumb pain only and none by the anticipation of toe pain only.
3. When comparing the cerebellar areas involved in pain experience and the anticipation of that experience, we found a relatively large area, mainly in Crus I and lobule VI on the contralateral side, that was activated by nociceptive stimulation as well as nociceptive anticipation, both for the thumb and for the toe. This indicates that the anticipation of a nociceptive stimulus is processed by the same cerebellar areas as the actual nociceptive stimulus and that this occurs for the thumb and toe alike, i.e., without a substantial somatotopic organization.

### General Aspects of the Experimental Setup and Analysis

In this study, we have used electrical stimulation, applied with a Neurometer [25, 26]. In rats, transcutaneous stimulation with 5-Hz sine waves stimulates mainly C-fibers [27, 28]. This suggests that this type of stimulation preferentially induces pain and not touch. Our subjects, who all declared that the stimuli induced a clear sensation of pain rather than an innocuous sensation like touch, corroborated this notion. We therefore conclude that our stimulation protocol preferentially activated nociceptive fibers.

A potential weakness of our study is that somatotopy was inferred based on the stimulation of only two body parts. This is especially true, since nociceptive stimulation may evoke confounding factors like perceived threat and unpleasantness that may differ between thumb pain and toe pain.

It may be argued that the present statistical analysis, which was chosen beforehand based on literature describing similar experiments and using similar group sizes in the human somatosensory cortex and cerebellum [9, 29], might be too liberal. An uncorrected statistical threshold of 0.005 in the cerebellum could indeed lead to an increase in false positives (type I errors). Future studies might consider using a stricter uncorrected threshold and/or voxel-based thresholding. These alternatives have, however, the drawback of an increased number of false negatives (type II errors), which might be a serious problem in experimental manipulations that affect the BOLD signal only slightly. Instead, we used a very conservative cluster-extent threshold of 24 voxels based on Coombes and Misra [9]. Alternatively, the present experiment may provide cerebellar regions that would allow future studies to use specific regions of interest rather than whole brain/whole cerebellum analyses, thereby drastically decreasing the number of voxels to be analyzed.

### The Activity Pattern of the Cerebellum During Nociceptive Thumb and Toe Stimulation

Nociceptive stimulation of the thumb and toe resulted in a clear activation of the pain matrix in the brain, including the insula, post central gyrus, cingulate gyrus, and cerebellum, which is in agreement with previous studies (see [30, 31] for review). Specifically, in the primary somatosensory cortex, we found an activation in the hand area after thumb stimulation and in the foot area after toe stimulation, as shown previously for hand and foot stimulation with capsaicin [32], electrical stimuli [33], or nociceptive laser stimulation [34]. These findings validated our experimental setup, allowing for a detailed analysis of the activation patterns in the cerebellum.

We have combined thumb and toe stimulation within one experiment, in order to keep circumstances of the thumb and toe stimulation, like arousal, attention, and visceral activation. This will allow for a comparison analysis in which it can be determined for each voxel, whether its activity is correlated more with toe than with thumb activation, or with both. These comparisons indicate that there is indeed a somatotopic organization of the cerebellum for nociceptive stimuli originating from different body parts. At this point, it should be stressed that the somatotopic organization of the cerebellum for sensory and motor input has been extensively studied in the past century and is still under much debate (see [35, 36] for review). With respect to fMRI studies, like the present study, results are mapped to cerebellar lobules, while the cerebellum is functionally organized in longitudinal zones that run across the various lobules. These zones cannot be reliably identified with fMRI, which may lead to a fractured (patchy) somatotopy [35]. Furthermore, in functional studies, it is often difficult to disentangle activities related to sensory input and (subsequent) motor output. This makes it difficult to compare the results of our study with other studies in detailed anatomical terms. Nevertheless, our finding that some areas in the cerebellum are activated by both thumb and toe nociceptive stimulation, while other areas are separately activated, suggesting a somatotopic organization, remains valid and should be interpreted in general terms. One subdivision from the literature, stating that medially located parts of the cerebellum are preferentially involved in basic sensory-motor performances, while cognitive tasks tend to engage lateral cerebellar regions, i.e., lobule VI and lobule VII (Crus I, Crus II, and VIIb) [37], seems in general agreement with our findings, since we find activations both medially and laterally in the cerebellum, as is to be expected when using pain stimuli which, by their nature, have both sensory-motor as well as emotional-cognitive aspects.

While the thumb and toe areas are separate, they are to a large extent located in the same lobules of the cerebellum, i.e., lobules VI and VIII of the posterior cerebellum and lobules IV–V in the anterior cerebellum. Crus II of the cerebellum is an exception as it is activated only by thumb stimulation. Activation of lobule VI has been reported by several other studies that have used nociceptive temperature stimulation [38, 39]. Lobules IV–VI have been described as being involved in sensory-motor processing, and lobule VIIIb in secondary sensory processing [40]. Thus, our findings suggest that the activation in the hemispheres of lobuli VI and VIII as well as lobules IV–V and Crus II is organized in a somatotopic manner, at least with respect to nociceptive stimulation of the thumb and the toe.

When the areas in the cerebellum that were activated by thumb as well as by toe stimulation were examined, we found that most clusters of activation were located in the vermis of lobuli IV–V, VI, VII, IX, and X; bilaterally in Crus I; and a small cluster in lobule VI ipsilaterally, i.e., mainly in the posterior cerebellum. This finding shows that nociceptive stimuli on the thumb and toe also activate areas of the cerebellum in a non-somatotopic manner. Activation of the vermis has also been shown in other studies [41] using peripheral nociceptive stimulation, although these studies have demonstrated activation primarily in the vermis of the anterior cerebellum, while we found that the bulk of the activation was located in the vermis of the posterior cerebellum. The posterior vermis has been described as the limbic part of the cerebellum [42]. It makes sense for nociceptive input, which is a highly emotional type of information, to reach the limbic area of the cerebellum. Crus I, which is considered part of the cognitive cerebellum [42], is also substantially activated by both the toe and the thumb. Thus, it seems that the somatotopically organized input to the cerebellum after nociceptive stimulation preferentially activates sensory-motor areas of the posterior cerebellum, while the same input also activates cognitive and limbic parts of the cerebellum in a non-somatotopic manner.

### The Activity Pattern of the Cerebellum During the Anticipation of Nociceptive Thumb and Toe Stimulation

Anticipation of thumb stimulation, but not anticipation of toe stimulation, activated limited areas of lobules VI and VIII ipsilaterally, as after the pain-only stimulation. Remarkably, the largest clusters of activation were found in the conjunction analysis, which seems to indicate that the anticipation of a nociceptive stimulus is a situation that the cerebellum is processing primarily in a non-somatotopic manner.

### Comparing Pain and the Anticipation of Pain

Anticipation of a nociceptive stimulus was first shown to lead to cerebellar activation by Ploghaus et al. [41]. They found that nociceptive heat stimuli, when compared to a warmth stimulus, localized bilaterally around the midline in the anterior cerebellum, while the anticipation of nociceptive heat localized ipsilaterally in the posterior cerebellum, thus showing differential localization of pain and its anticipation within the cerebellum. In contrast, our conjunction analysis makes clear that the areas in the cerebellum that were activated by an actual pain stimulus are also activated by the anticipation of such a stimulus and that these areas, i.e., Crus I and lobule VI, were located bilaterally in the posterior cerebellum. This is in accordance with findings in other areas of the brain, both at the cortical and at the subcortical level, which showed that the anticipation of pain was found to activate both the same areas as activated during actual pain perception as well as other areas, possibly involved in preparing for the expected nociceptive stimulus [43]. Furthermore, a study [44] comparing activations in the cerebellum by aversive pictures with activations of a heat pain stimuli also showed that Crus I and lobule VI (and additionally lobule VIIb) were activated by both stimuli. Our finding that Crus I and lobule VI become activated by both the anticipation of a specific pain stimulus and the actual stimulus, irrespective of the localization of that stimulus, would fit very well with the idea that these cerebellar areas are involved in processing general aversive emotions, like pain. Interestingly, emotions like (the expectation of) reward, which are on the opposite side of aversion in the emotional spectrum, are also processed in the cerebellum [45, 46]. Recordings from granule cells in lobules V and VI of the mouse cerebellum showed an expectation of reward-related activity even independent of any (preparatory) motor activity [46].

In conclusion, many fMRI studies have shown activation of the cerebellum in pain processing [47]. In these studies, it remained unclear how the nociceptive information reaches the cerebellum. Anatomical and physiological data show that the cerebellum receives this information directly, through connections with the spinal cord, as well as indirectly through its connections with the cortex. It seems likely that the input that is organized in a somatotopical manner reflects the direct input from the spinal cord, while the non-somatotopically activated parts of the cerebellum receive more general contextual information, like (expected) nociception, indirectly through cortical and subcortical connections. These parts are possibly involved in processing general emotions, like aversion and reward, thus allowing (expected) emotional states to affect sensory-motor processing in the cerebellum. The findings in this study seem to support this notion.

## Electronic Supplementary Material

Table S1. Group analysis of activation induced by nociceptive stimulation of thumb and toe. Cluster size (Cluster) in voxels, T max (T) and its MNI coordinates (x, y, z) and side at which the activation occurs (Side) are given followed by the specification of the number of voxels (N voxels) within that cluster. Significant areas with an uncorrected threshold of *p*=0.001 and a cluster-extent threshold of at least 104 voxels are shown in italic, while areas containing sub-threshold clusters are shown in regular face (XLSX 12 kb)

Table S2. Group analysis of differences in activation in the cerebellum of nociceptive stimulation after the anticipation of pain of thumb and toe. Cluster size (Cluster) in voxels, T max (T) and its MNI coordinates (x, y, z) and side at which the activation occurs (Side) are given followed by the specification of the number of voxels (N voxels) per structure (Structure) within that cluster. Significant areas with an uncorrected threshold of *p*=0.005 and a cluster-extent threshold of at least 24 voxels are shown in italic, while areas containing sub-threshold clusters are shown in regular face (*n*=14) (XLS 24 kb)
